# Comprehensive mutation profiling and mRNA expression analysis in atypical chronic myeloid leukemia in comparison with chronic myelomonocytic leukemia

**DOI:** 10.1002/cam4.1946

**Published:** 2019-01-11

**Authors:** Muhammad Faisal, Helge Stark, Guntram Büsche, Jerome Schlue, Kristin Teiken, Hans H. Kreipe, Ulrich Lehmann, Stephan Bartels

**Affiliations:** ^1^ Institute of Pathology Hannover Medical School Hannover Germany

**Keywords:** aCML, CMML, machine learning algorithm, nCounter, NGS

## Abstract

Atypical chronic myeloid leukemia (aCML) and chronic myelomonocytic leukemia (CMML) represent two histologically and clinically overlapping myelodysplastic/myeloproliferative neoplasms. Also the mutational landscapes of both entities show congruencies. We analyzed and compared an aCML cohort (n = 26) and a CMML cohort (n = 59) by next‐generation sequencing of 25 genes and by an nCounter approach for differential expression in 107 genes. Significant differences were found with regard to the mutation frequency of *TET2*, *SETBP1*, and *CSF3R*. Blast content of the bone marrow revealed an inverse correlation with the mutation status of *SETBP1* in aCML and *TET2* in CMML, respectively. By linear discriminant analysis, a mutation‐based machine learning algorithm was generated which placed 19/26 aCML cases (73%) and 54/59 (92%) CMML cases into the correct category. After multiple correction, differential mRNA expression could be detected between both cohorts in a subset of genes (*FLT3*, *CSF3R*, and *SETBP1* showed the strongest correlation). However, due to high variances in the mRNA expression, the potential utility for the clinic is limited. We conclude that a medium‐sized NGS panel provides a valuable assistance for the correct classification of aCML and CMML.

## 1 INTRODUCTION

Atypical chronic myeloid leukemia (aCML) and chronic myelomonocytic leukemia (CMML) represent two entities of hematopoietic neoplasms which display a combination of myelodysplastic and myeloproliferative features (MDS/MPN).^1^

Atypical CML is a rare *BCR‐ABL1* negative disease with an estimated frequency of 1 to 2 cases for every 100 CML cases.^2^ World health organization (WHO) criteria for aCML disease are (a) absence of *BCR‐ABL1* rearrangement, (b) >13 000 leukocytes, and (c) <10% monocytes.^1^ The median overall survival (OS) is 25 months, approximately 40% of aCML cases show leukemic transformation within 18 months of diagnosis (source). Risk factors that impair OS in aCML patients are >65 years, female gender, <10 g/dL hemoglobin, leukocytes >50 × 10^9^ L, and circulating immature myeloid cells.^2^, ^3^

During the last decade, the establishment of genetic markers significantly improved diagnosis of myeloid neoplasms. *CSF3R* mutations, which were discovered in 2013, are shared by aCML and chronic neutrophilic leukemia, a rare subtype of myeloproliferative neoplasms (MPN) characterized by neutrophilia with less than 10% immature precursors in the peripheral blood.^1^, ^4^, ^5^ *SETBP1* mutations are also found to be closely related to the aCML phenotype. Mutations are present in 24% to 33% of cases and are associated with leukocytosis and shorter OS.^6^, ^7^ Furthermore, *ETNK1* gene mutations were found recently in a minority of cases of aCML and CMML.^9^, ^10^ Mutational landscape of CMML are already analyzed in large series of patient samples.^11^, ^12^ *ASXL1*, *SRSF2*, and *TET2* gene mutations are found frequently in CMML, each in approximately 40% of cases.^14^ However, none of these mutations are specific for aCML or CMML, even *SETBP1* mutations can be found in a subset of CMML cases.^15^

Bone marrow histopathology reveals tremendous overlap between aCML and CMML which sometimes have very similar presentation. The WHO classification separates both myelodysplastic‐myeloproliferative neoplasm according to peripheral blood findings (10% blood monocytes and ≥1000 monocytes/µL for CMML, >10% immature granulocytic precursors in aCML), although monocytosis in the context of neutrophilia may be present in both diseases.^16^ In addition, leukocytosis may also be found in the peripheral blood of CMML patients not only in case of aCML disease.^14^

Here we report a comprehensive mutation profiling (by next‐generation sequencing, NGS) and mRNA expression analysis (by nCounter technology) of aCML vs CMML patient sample cohorts. Our aim was to delineate overlapping and discriminatory molecular features between both entities. To the best of our knowledge, high throughput mRNA expression profiling of bone marrow cells in aCML was not performed before.

## 2 PATIENT SAMPLES, MATERIALS, AND METHODS

Decalcified formalin‐fixed, paraffin‐embedded (FFPE) bone marrow trephines were selected retrospectively from the archive of the Institute of Pathology, Hannover Medical School, Germany. All cases were analyzed in the routine diagnostic procedures of the institute. In total, 119 patient samples (aCML n = 26, CMML n = 59, reactive controls n = 34) were included and clinical data (age, gender, hemoglobin, leukocyte count, percentage of monocytes, and blasts in peripheral blood) were collected, if available. Disease classification was following the WHO criteria.^1^

Reactive controls were obtained from individuals who underwent a bone marrow biopsy, but histomorphological examination was unobtrusive. All samples of this control cohort were analyzed by sequencing as described below. None of the 25 genes under investigation showed a gene mutation.

Statistical analysis was performed with GraphPad Prism Version 5.00, two‐sided Fisher’s exact test (results are considered to be statistically significant when *α* < 0.05) and two‐tailed Mann‐Whitney *U* test (results are considered to be statistically significant when *P* < 0.05) were used. The study design is following the guidelines of the Hannover Medical School ethics committee. Informed consent was obtained from all patients under study.

### 2.1 Nucleic acid extraction

Extraction of DNA and RNA was performed with the Maxwell RSC instrument (Promega, Madison, WI, USA) according to the manufacturer's recommendations. Three to five sections of 10 µm each were taken, depending on the size of the trephine. DNA was extracted with the Maxwell RSC DNA FFPE kit; RNA was extracted with the Maxwell RSC RNA FFPE kit (Promega). Nucleic acid concentration were quantified using a Qubit 2.0 fluorometer (Invitrogen, Darmstadt, Germany) and the Qubit dsDNA high sensitivity kit as well as the Qubit RNA high sensitivity assay kit (LifeTechnologies, Carlsbad, CA, USA).

### 2.2 NGS

Targeted re‐sequencing of 23 genes was performed with a customized panel as described previously.^17^ *MPL* Exon 10 and *ETNK1* Exon 3 sequencing were performed by Pyrosequencing as described.^18^ PCR and sequencing primers for pyrosequencing are listed in Table S1. Data evaluation and variant annotation were performed with the ANNOVAR software and database tools^19^ (http://annovar.openbioinformatics.org/en/latest/).

### 2.3 *BCR‐ABL1* fusion gene analysis

Multiplex reverse transcription PCR to detect *BCR‐ABL1* fusion transcripts was performed as described.^20^ In the entire aCML cohort (n = 26), the *BCR‐ABL1* fusion transcript was absent.

### 2.4 Classification of samples

Classification analysis was performed in the R programming language (https://www.r-project.org/) using logistic regression (R package stats), multinomial logistic regression (R package nnet), linear discriminant analysis (R package MASS), random forest analysis (R package randomForest), and support vector machines with linear kernel (R package e1071). Except for random forest analysis, for each possible number of genes used for classification, the most suitable gene subset was identified using an exact leaps‐and‐bounds algorithm optimizing the Tau‐squared coefficient (R package subselect). This algorithm also automatically excluded gene sets with highly correlated variables. The performance all resulting models for unknown samples was estimated using leave‐one‐out cross‐validation (R package caret).

### 2.5 nCounter‐based quantification of mRNA expression

For mRNA expression analysis, 150 ng of total RNA was used per patient sample. A customized code set was designed including 107 genes (Table S2) which are involved in cell signaling, transcription, apoptosis and mitosis, epigenetic regulation, and RNA processing. Hybridization of RNA and probes was performed using the nCounter Gene Expression Assay according to the manufacturer's recommendations (NanoString Technologies, Seattle, WA, USA). Processing and analysis of mRNA expression data was performed using the nSolver™ Analysis Software version 3.0, the nCounter™ Advanced Analysis Package version 1.1.5 as well as in‐house software written in the R programming language. Normalization parameters included background normalization (arithmetic mean of reactive controls), positive control normalization (geometric mean of positive controls), and reference gene normalization. We used the GeNorm algorithm^21^ included in the nCounter™ Software to automatically select the five most suitable reference genes for normalization (*EHMT1*,* HDAC3*, *PIAS1*, *NUBP1*, and* PIK3R4*). After normalization, samples that exhibited less than 20 counts for more than 50% of all mRNA targets were excluded from further analysis. Statistical analysis included comparisons of multiple groups using the Kruskal‐Wallis test with correction for multiple testing (Benjamini‐Hochberg) as well as pairwise comparisons against reference samples using the Mann‐Whitney test with correction for multiple testing (Benjamini‐Hochberg). The reactive control sample cohort (n = 34) was used as the normal reference for mRNA expression.

## 3 RESULTS

### 3.1 Clinical characteristics of patient cohorts

Clinical characteristics of cases are summarized in Table 1. Mean values of gender, age, and hemoglobin are not different between the cohorts. Individual data of all patients under study are listed in Tables S3 and S4. As expected, the mean leukocyte count in the aCML cohort was significantly higher than in the CMML cohort (52.1 × 10^3^ vs 25.4 × 10^3^, *P* < 0.0001). Likewise, the percentage of monocytes in the peripheral blood is highly increased in the CMML cohort (21.1% vs 5.3%, *P* < 0.0001). Excess of blasts in the bone marrow was detectable in 53.8% of the aCML and 39.0% of the CMML cases. Seven cases (two aCML and five CMML) were in transformation into an acute myeloid leukemia and had blast counts of approximately 20% (Tables S3 and S4).

**Table 1:** Clinical data of the aCML and CMML patients

	aCML (n = 26)	CMML (n = 59)	*P*‐value‡,¥
Gender	73.1% male	67.8% male	ns‡
Age	72.3 y (range 46‐89; SD 11.7)	74.9 y (range 26‐90; SD 9.6)	ns‡
Hemoglobin	10.1 g/dL (range 6.1‐14.3; SD 2.5)	10.6 g/dL (range 5.0‐15.0; SD 2.3)	ns‡
Leukocytes	52.1 × 10^3^ (range 15.7‐300.0; SD 60.0)	25.4 × 10^3^ (range 0.7‐234.5; SD 47.0)	*** (*P* < 0.0001)‡
Monocytes	5.0% (range 1.0‐10.0; SD 3.5)	21.5% (range 2.0‐42.0; SD 10.6)^a^	*** (*P* = 0.0003)‡
Blasts in peripheral blood	14 (53.8%)	23 (39.0%)	ns¥

ns, not significant (*P* > 0.05); SD, standard deviation.

a Available biopsies of CMML patients sometimes show lower absolute monocyte counts than 1000 due to therapeutic effects (see Table S4). At the time of diagnosis, absolute monocyte count was above 1 × 10^9^/L, regarding to the WHO criteria.

^‡^ Mann‐Whitney test.

^¥^ Fishers‐exact test.

### 3.2 Mutation profiling of aCML and CMML

**Figure 1:**
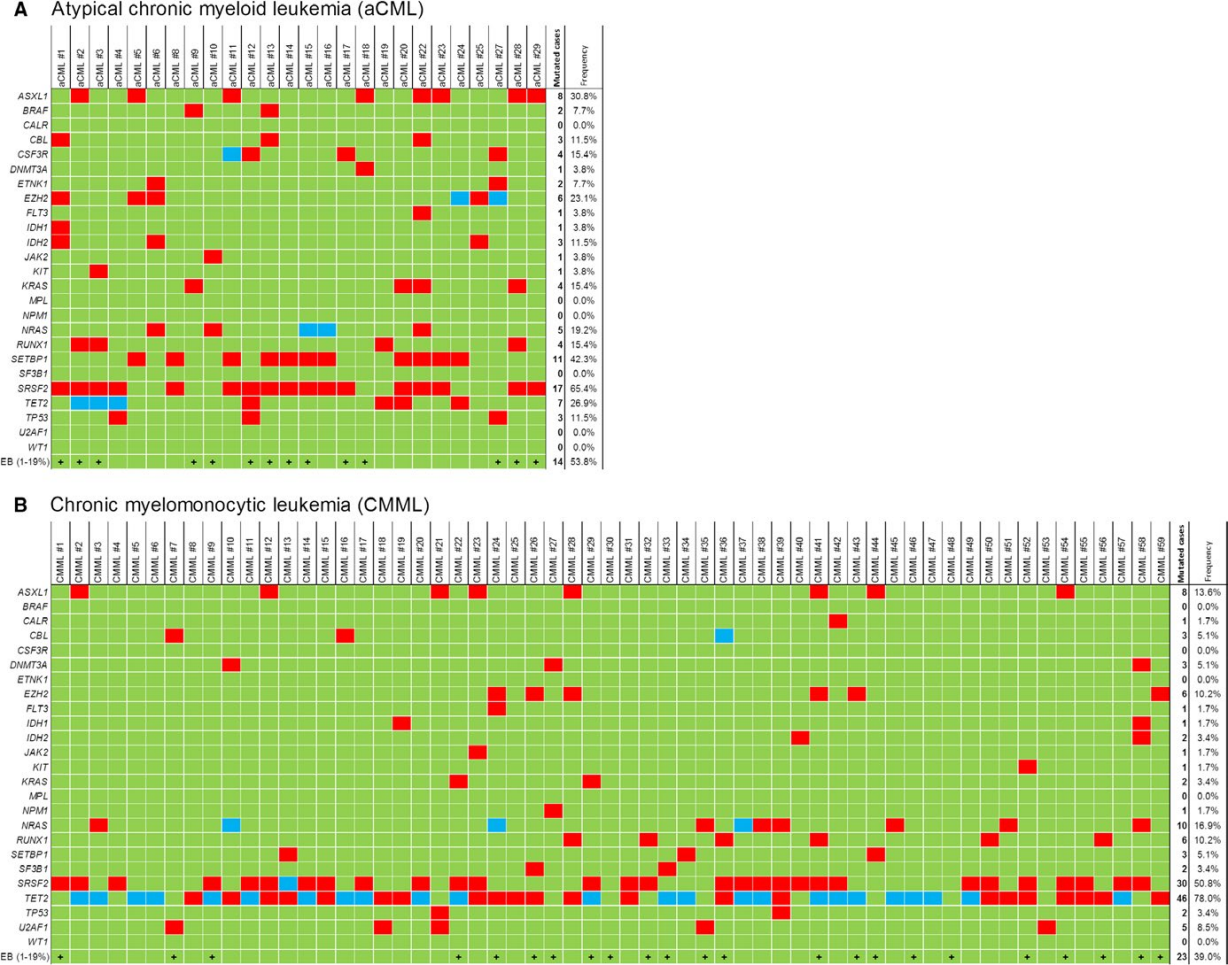
Results of mutation profiling in the aCML cohort (A, n = 26) and in the CMML cohort (B, n = 59). Green‐filled squares indicate a wild‐type, red‐filled squares indicate one pathogenic gene mutation, and blue‐filled squares indicate two pathogenic mutations within the specific gene. Excess of blasts in the bone marrow (EB) is indicated with + (see Tables S3 and S4 for patient details)

Results of the comprehensive mutational profiling in 25 genes are shown in Figure 1. In the aCML cohort in total 92 pathogenic mutations could be detected. The CMML cases show 164 pathogenic mutations in the genes under investigation. Mean number of gene mutations were significantly different between the cohorts (3.54 in aCML vs 2.78 in CMML; *P* = 0.0266; Table 2). This effect was even stronger when several mutations in the same gene were excluded from the calculation (3.23 vs 2.29; *P* = 0.0009).

**Table 2:** Comparison of the mutational profiling results between the cohorts

	aCML (n = 26)	CMML (n = 59)	*P*‐value‡,¥
Mean no. of mutated genes per case	3.23 (range 2‐7; SD 1.21)	2.29 (range 0‐5; SD 1.05)	*** (*P* = 0.0009)‡
Mean no. of mutations per case	3.54 (range 2‐7; SD 1.36)	2.78 (range 0‐6; SD 1.23)	* (*P* = 0.0266)‡
*TET2*	7 (26.9%)	46 (78.0%)	*** (*P* < 0.0001)¥
*SETBP1*	11 (42.3%)	3 (5.1%)	*** (*P* < 0.0001)¥
*CSF3R*	4 (15.4%)	0 (0.0%)	** (*P* = 0.0074)¥
*ASXL1*	8 (30.8%)	8 (13.6%)	ns¥
*EZH2*	6 (23.1%)	6 (10.2%)	ns¥
*KRAS/NRAS/BRAF*	9 (34.6%)	12 (20.3%)	ns¥
*IDH1/IDH2*	4 (15.4%)	3 (5.1%)	ns¥
*SRSF2*	17 (65.4%)	30 (50.8%)	ns¥

ns, not significant (*P* > 0.05); SD, standard deviation.

^‡^ Mann‐Whitney test.

^¥^ Fishers‐exact test.

Nine patients in the aCML cohort showed two gene mutations, 17 patients (65.4%) revealed three or more gene mutations (Figure 1A). With over 65%, *SRSF2* gene mutations represent the most frequent genetic lesion in these aCML patients. Furthermore, 11 patients showed mutation of *SETBP1 *(42.3%), and nine cases of these were *SRSF2/SETBP1 *co‐mutated. Taken together, *KRAS*, *NRAS *and/or *BRAF* are mutated in 9/26 patients (34.6%). *ASXL1* and *EZH2* were found to be mutated in eight and six cases (30.8% and 23.1%), respectively. Interestingly, only in one of these cases both genes are co‐mutated (aCML#5, Figure 1A). Consequently, half of the aCML patients displayed a defect in the histone modification (Table S3).

In the CMML cohort, a high frequency of *TET2* (78.0%) and *SRSF2* mutations (50.8%) was detectable (Figure 1B). Only eight of the 59 cases (13.6%) exhibit neither a *TET2* nor a *SRSF2* mutation. The presence of splice factor mutations in CMML is even higher when the five *U2AF1* and two *SF3B1* mutations were taken into account. Splice factor genes are mutated exclusively in our cohort; consequently 37 of 59 (62.7%) CMML patients harbor a defect in the spliceosome. However, none of the additionally analyzed genes alone showed mutation frequencies above 20% of the cases. Combined, *KRAS* and *NRAS* are mutated in 12 (20.3%) of all CMML cases. *ASXL1* (8/59, 13.6%) and *RUNX1* mutations (6/59, 10.2%) were less frequently mutated than in other studies.^13^, ^14^

When the mutation profiling results of both cohorts are compared, a different mutational landscape is distinguishable (Table 1B). *TET2* mutations are specific for CMML (*P* < 0.0001), whereas *SETBP1* and *CSF3R* mutations are significantly more frequent in aCML (*P* < 0.0001, and *P* = 0.0074, respectively). *IDH*, *RAS*, *ASXL1*, and* SRSF2* mutations are more frequent in aCML, but the differences are not significant.

Table 3 shows the frequently mutated genes in the aCML cohort related to the presence of increased blasts counts in the bone marrow, independently of the blast percentage. None of the genes showed a correlation with the blast excess, except for *SETBP1*. In the aCML cohort, the presence of a *SETBP1* gene mutation showed a significant correlation with the absence of blast increase (*P* = 0.0447).

**Table 3:** Frequently detected gene mutations in the aCML cohort in comparison with blast excess in the patient samples

Mutated genes	aCML EB+ (n = 14)	aCML EB‐ (n = 12)	*P*‐value¥
*SETBP1*	3 (21.4%)	8 (66.6%)	* (*P* = 0.0447)
*EZH2*	2 (14.3%)	4 (33.3%)	ns
*TET2*	3 (21.4%)	4 (33.3%)	ns
*SRSF2*	10 (71.4%)	7 (58.3%)	ns
*ASXL1*	4 (28.6%)	4 (33.3%)	ns
*KRAS/NRAS/BRAF*	5 (35.7%)	4 (33.3%)	ns

EB, excess of blasts in the bone marrow; ns, not significant (*P* > 0.05).

^¥^ Fishers‐exact test.

When related to the presence of blast increase in the bone marrow of CMML patients, only *TET2* displayed a significant association. Patients with *TET2* mutations had a lower risk of increased blast counts (*P* = 0.0218; Table 4). Interestingly, of the 14 patients which showed blast increase and *TET2* mutation, only five patients exhibited an isolated *TET2* mutation. The remaining nine samples harbored *TET2*/splice factor co‐mutations (Figure 1B and Table S4).

**Table 4:** Frequently detected gene mutations in the CMML cohort in comparison with blast excess in the patient samples

Mutated genes	CMML EB+ (n = 23)	CMML EB− (n = 36)	*P*‐value¥
*TET2*	14 (60.9%)	32 (88.8%)	* (*P* = 0.0218)
*RUNX1*	4 (17.4%)	2 (5.5%)	ns
*SRSF2/U2AF1/SF3B1*	14 (60.9%)	20 (63.9%)	ns
*KRAS/NRAS*	5 (21.7%)	7 (19.4%)	ns
*ASXL1*	3 (13.0%)	5 (13.9%)	ns

EB, excess of blasts in the bone marrow; ns, not significant (*P* > 0.05).

^¥^ Fishers‐exact test.

### 3.3 Classification of samples via mutational profiling

**Figure 2:**
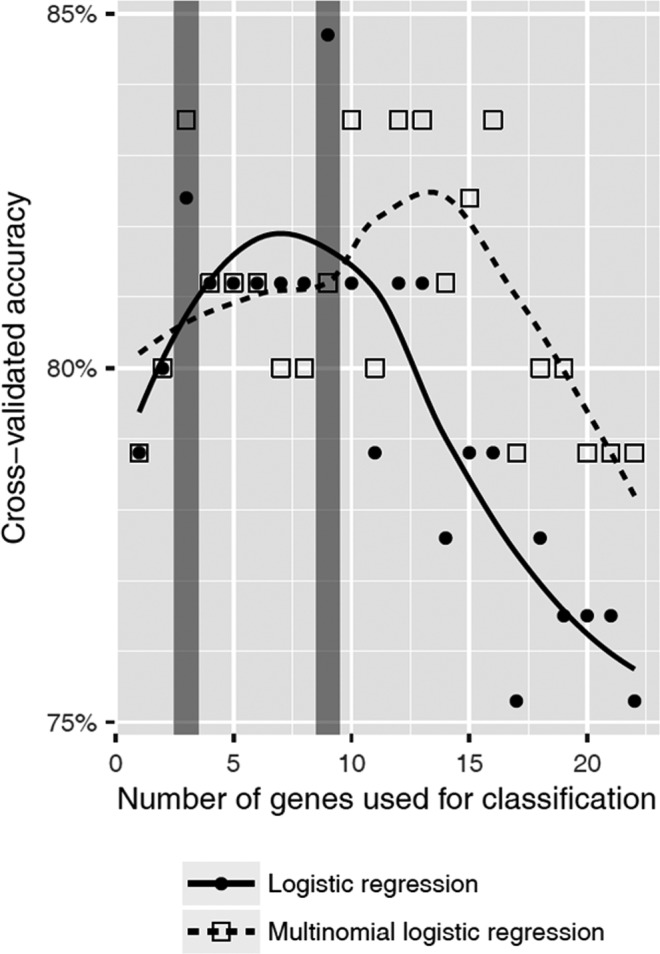
Accuracy of machine learning algorithms for the prediction of aCML and CMML via gene mutational frequency. Using the mutational frequency of 25 selected genes, logistic regression and multinomial logistic regression performed best at predicting the class membership of samples to either aCML or CMML among a variety of tested algorithms. The accuracy of all models was evaluated via leave‐one‐out cross‐validation, since all samples have to be used for the training of models. In order to prevent overfitting, the number and subsets of genes used for classification were selected using an exact leaps‐and‐bounds algorithm. Optimal accuracy for multinomial logistic regression is achieved when using a subset of three genes (*SETBP1*, *TET2*, and *CSF3R*, 84% accuracy) or a subset of nine genes for logistic regression (*SETBP1*, *TET2*, *CSF3R*, *TP53*, *U2AF1*, *RUNX1*, *KRAS*, *IDH2*, and *BRAF*, 85% accuracy, Figure S1)

In order to assess whether the frequency of mutations in specific genes can be used to classify unknown samples into one of the two disease groups, we performed classification analysis using the mutation profiles shown in Figure 1A,B. Of the five different classification algorithms used in this work, logistic regression and multinomial logistic regression yield the highest accuracy during cross‐validation. The highest estimated accuracy for unknown samples (85%) was achieved using the frequency of mutations of the nine genes *SETBP1*, *TET2*, *CSF3R*, *TP53*, *U2AF1*, *RUNX1*, *KRAS*, and *IDH2* (Table S1; Figure 2). The best model achieved correct classification for 19 of the 26 samples from the aCML cohort (73%) and 54 of 59 samples from the CMML cohort (92%).

### 3.4 Comprehensive mRNA profiling

**Figure 3:**
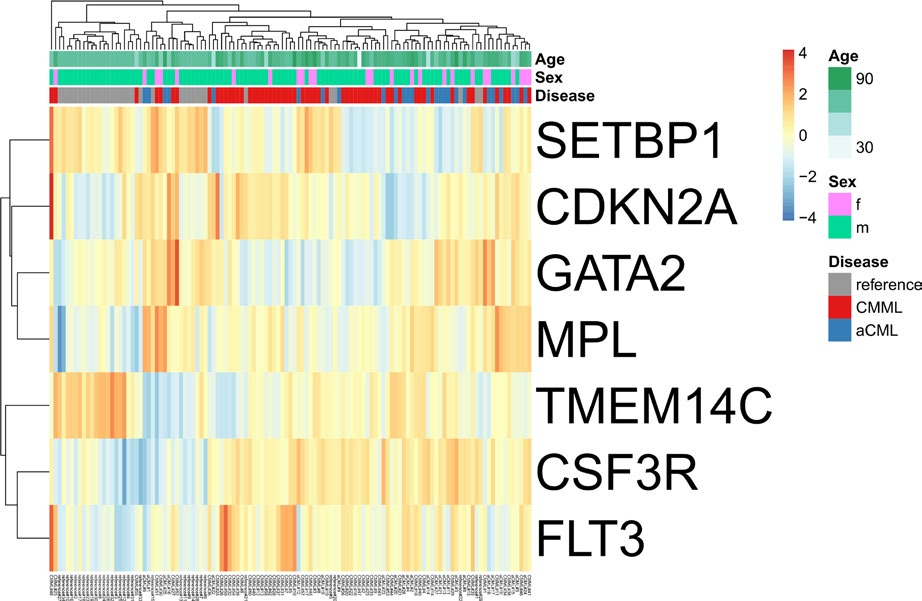
Centered, scaled, and hierarchically clustered log2 mRNA expression of statistically different expressed genes of all samples investigated in this study. In total, 107 genes were investigated for mRNA expression level in the aCML (n = 26) and CMML (n = 59) cohort as well as reference samples (n = 34, FFPE bone marrow samples from individuals with reactive histomorphology). Genes *IKZF1*, *U2AF1*, *U2AF2*, *UPF3B*, *USP39*, *WAC*, *WT1*, *ZNF143*, *ZNF346*, and *ZRSR2* were excluded from further analysis due to constantly low counts

The analysis of mRNA expression of all 86 samples (Figure 3) revealed statistically significant changes in the expression levels of seven genes after correction for multiple testing between individuals with morphologic features of reactive states (reference) as well as the aCML and CMML cohorts. This applies to the comparison of multiple groups as well as pairwise comparisons of both patient cohorts against the reference. Figure S2 shows the seven genes with significant differences after correction for multiple testing (Benjamini‐Hochberg). The highest differences exhibited *FLT3* (highest expression in CMML compared to aCML and reference samples), *CSF3R *(higher expression in aCML and CMML compared to reference samples), and *SETBP1* (lower in aCML and CMML compared with the reference samples). However, even these genes show only log2 differences of 2, meaning four times higher or lower relative expression. Furthermore, high variances and many outliers are observable.

## 4 DISCUSSION

### 4.1 Mutational landscape of aCML and CMML

Atypical CML represents a rare disease subtype of the MDS/MPN category^1^; therefore, information about the mutational landscape in extended cohorts is limited so far. In a study of 25 aCML cases, Patnaik et al^3^ performed mutational profiling of 29 genes. Surprisingly, when comparing this aCML cohort with the 26 cases in our study some results differ relating to the frequency of mutated genes (Table 5). Similar to our study, Patnaik et al could detect gene mutations in all cases. *ASXL1* is the most frequently mutated gene in their study, detected in 28% (7/25) of the patients. We could show a similar *ASXL1* mutation frequency in 30.8% (8/26) of our cases; all variants represented frameshift or nonsense mutations. Obviously, the frequency of *SRSF2* mutations is different with 65.4% (17/26) in our aCML cohort in comparison with 12% (3/25) in the study of Patnaik et al Furthermore, *SETBP1* shows different mutation frequency in both cohorts (11/26; 42.3% vs 3/25, 12%). Probably, these differences resulted partly from the limited number of cases in both studies. However, it is high likely that aCML cases do not represent a homogenous disease entity. In particular, the differences in *SETBP1* mutation frequency between both studies are interesting, because we could show that the presence of these mutations is inversely correlated with the presence of blast increase in the bone marrow. Unfortunately, follow‐up data in our cohort are only available in a minority of cases, not sufficient for a prospective calculation of risk factors for progression.

**Table 5:** Comparison of the frequently mutated genes between the aCML cohort in the present study with a published cohort, similar in number of cases and analyzed genes

Samples	Patnaik et al^3^	Present study (aCML)	*P*‐value¥
	n = 25	n = 26	
Genes	29	25	
*ASXL1*	7/25 (28%)	8/26 (30.8.6%)	ns
*TET2*	4/25 (16%)	7/26 (26.9%)	ns
*NRAS*	4/25 (16%)	5/26 (19.2%)	ns
*CSF3R*	2/25 (8%)	4/26 (15.4%)	ns
*EZH2*	2/25 (8%)	6/26 (23.1%)	ns
*SETPB1*	3/25 (12%)	11/26 (42.3%)	* (*P* = 0.0266)
*SRSF2*	3/25 (12%)	17/26 (65.4%)	*** (*P* < 0.0001)

ns, not significant.

^¥^ *P*‐values were calculated by Fishers‐exact test.

Mutation profiling results of our CMML cohort are well comparable with previous studies. Most prominent mutated genes are *TET2* (78.0%), *SRSF2* (50.8%), *KRAS/NRAS *(20.3%), and *ASXL1 *(13.6%). Only *TET2* mutations seemed to be enriched in our study in comparison with 46%‐60% in the literature, whereas *ASXL1* is underrepresented in comparison with 26%‐40% literature values.^13^, ^14^ The presence of a *TET2* mutations is inversely correlated with the presence of blast increase in the bone marrow in the CMML cohort (*P* = 0.0218). This finding is well in line with an adversely impacted survival found in CMML patients with absent TET2 mutations.^22^

A machine learning classification approach using linear discriminant analysis revealed that the mutation profiles of aCML and CMML cases are apparently divergent enough to allow for classification of unknown samples using only nine gene loci with relatively high accuracy (>85%). The higher accuracy for the classification of CMML samples (92%) observed during this analysis is probably due to the higher sample size of the training set, indicating that further data might enable the generation of more precise and complex models and thus further facilitate the discrimination between both disease types. Machine learning approaches represent a possible feature to assist the histomorphological diagnosis in the era of digital pathology.

Nevertheless, it has to be kept in mind that MPN can undergo a progress or a transition into another disease type via clonal evolution resulting in acquisition of additional mutations or loss of genetic alterations. This phenomenon requires the investigation of regular follow‐up biopsies.^23^, ^24^

### 4.2 mRNA expression in aCML and CMML

Statistically significant differences in mRNA expression of seven genes could be observed between the aCML and CMML compared to reference samples for the 107 genes investigated in this work (Figure 3). The strongest effect in differential expression is detectable in *FLT3*, *SETBP1*, and *CSF3R* (Table S2). This is congruent with the significantly different mutation frequency for SETBP1 and CSF3R between aCML and CMML. However, high variances and many outliers in the mRNA count are observable. Unfortunately, this expression dataset could not contribute to a refining of the classification algorithm.

Future transcriptomics research on both disease entities should focus on other gene loci. It cannot be excluded that alternative splicing events, due to the high frequency of splice factor mutations in both cohorts, will cause a bias in our expression data. All expression counts for the specific mRNA based only on one probe per gene. Maybe alterative splicing, for example, increased exon skipping, has an influence on our results.

## 5 CONCLUSIONS

Mutation profiling reveals overlap and differences between aCML and CMML. Whereas *TET2* mutations are significantly enriched in CMML; *SETBP1* and *CSF3R* are more frequently but not exclusively mutated in aCML. For mutation profiling, targeted NGS panel represents an optimal approach with respect to costs and turn‐around‐time. It has been shown that sequencing studies of nine genes can identify the clonal abnormality in >90% of CMML cases.^11^ Consequently, whole exome sequencing studies found a highly similar number and frequency of gene mutations compared to the more restricted targeted NGS approach with 20 to 30 genes.^14^, ^25^, ^26^

In the approach presented, with 107 mRNA targets for expression analysis, statistically significant differences between the aCML and the CMML group could be detected after a multiple correction. However, our approach did not yield discriminatory markers for differential diagnostics of individual cases, because all alterations were shared by both diseases.

In our study, 18/26 (69%) aCML and 51/59 (86%) CMML cases harbor at least one mutation in the epigenetic modifier genes *TET2*, *IDH1/2*, *DNMT3A*, *EZH2*, and *ASXL1 *(Figure 1A,B). Consequently, alterations in the epigenetic regulation of aCML and CMML represent an interesting field for further studies.

## CONFLICT OF INTEREST

None declared.

## AUTHOR CONTRIBUTIONS

FM and SB carried out molecular analyses. FM, HS, and SB analyzed the data. GB, JS, KT, and HHK assessed histomorphological characteristics. HS, HHK, UL, and SB conceived the study wrote the manuscript.
