# Supplementary figures and images for: Comprehensive mutation profiling and mRNA expression analysis in atypical chronic myeloid leukemia in comparison with chronic myelomonocytic leukemia
Source: Cancer Med. 2019 Jan 11;8(2):742–50. doi: 10.1002/cam4.1946 (PMC6382710; doi:10.1002/cam4.1946)

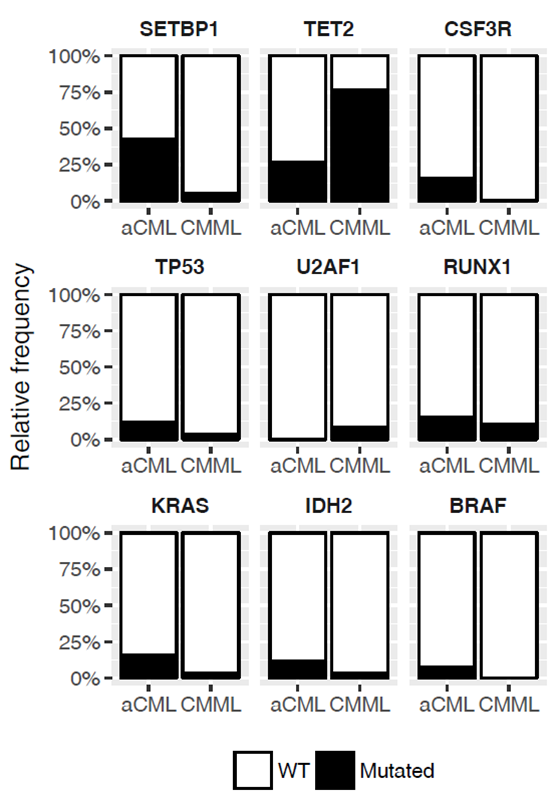

Supplement: Supplementary file 1 [file CAM4-8-742-s001.tif]

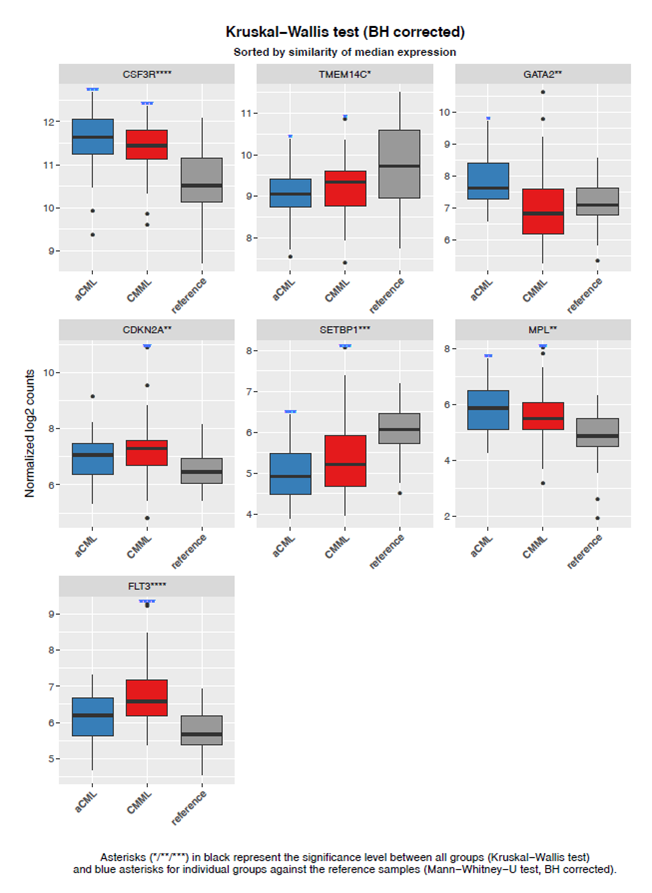

Supplement: Supplementary file 2 [file CAM4-8-742-s002.tif]
