# Supplementary material for: Comprehensive mutation profiling and mRNA expression analysis in atypical chronic myeloid leukemia in comparison with chronic myelomonocytic leukemia
Source: Cancer Med. 2019 Jan 11;8(2):742–50. doi: 10.1002/cam4.1946 (PMC6382710; doi:10.1002/cam4.1946)
Supplement: Supplementary file 5 [file CAM4-8-742-s005.docx]

Supplementary Table S3 Available clinical data and mutation profiling results of the aCML patient sample cohort (n=26)

| **Patient No.** | **Sex** | **Age** | **Hemoglobin** | **Leucocytes** | **Monocytes** | **Blasts** | **Gene mutations** | **coding** | **protein** | **allelic frequency** | **Comment** |
| --- | --- | --- | --- | --- | --- | --- | --- | --- | --- | --- | --- |
| aCML#1 | M | 67 | 8.0 g/dl | 64.0 x 10^3^ | 10.0% | <5% | *CBL* | c.11112A>G | p.Y371C | 27.0% |  |
|  |  |  |  |  |  |  | *EZH2* | c.2027G>C | p.R676T | 20.3% |  |
|  |  |  |  |  |  |  | *IDH1* | c.395G>A | p.R132H | 20.6% |  |
|  |  |  |  |  |  |  | *IDH2* | c.419G>A | p.R140Q | 21.1% |  |
|  |  |  |  |  |  |  | *SRSF2* | c.278_301del | p.P95_R102del | 18.3% |  |
| aCML#2 | W | 85 | 8.4 g/dl | 26.0 x 10^3^ | 2.0% | >5% | *ASXL1* | c.2757dupA | p.P920Tfs*4 | 43.1% |  |
|  |  |  |  |  |  |  | *SRSF2* | c.284C>A | p.P95H | 49.7% |  |
|  |  |  |  |  |  |  | *RUNX1* | c.493G>A | p.G165S | 39.0% |  |
|  |  |  |  |  |  |  | *TET2* | c.1918C>T | p.Q640* | 44.9% |  |
|  |  |  |  |  |  |  | *TET2* | c.3979C>T | p.Q1327* | 41.0% |  |
| aCML#3 | M | 89 | 9.9 g/dl | 23.8 x 10^3^ | 4.0% | >5% | *KIT* | c.2447A>T | p.D816V | 34.5% | associated MF1 |
|  |  |  |  |  |  |  | *RUNX1* | c.493G>T | p.G165C | 36.9% | associated systemic |
|  |  |  |  |  |  |  | *SRSF2* | c.284C>G | p.P95R | 45.3% | mastocytosis |
|  |  |  |  |  |  |  | *TET2* | c.1693_1697del | p.R565Qfs*21 | 9.4% |  |
|  |  |  |  |  |  |  | *TET2* | c.2230C>T | p.Q744* | 50.1% |  |
| aCML#4 | M | 81 | 8.8 g/dl | 20.0 x 10^3^ | 10.0% |  | *SRSF2* | c.284C>G | p.P95R | 49.6% |  |
|  |  |  |  |  |  |  | *TET2* | c.1985_1986del | p.G662Afs*39 | 11.7% |  |
|  |  |  |  |  |  |  | *TET2* | c.2148insA | p.M716Ifs*17 | 66.7% |  |
|  |  |  |  |  |  |  | *TP53* | c.730G>C | p.G244R | 51.9% |  |
| aCML#5 | M | 58 | 9.7 g/dl | 300.0 x 10^3^ | 6.0% |  | *ASXL1* | c.1773C>A | p.Y591* | 31.2% |  |
|  |  |  |  |  |  |  | *EZH2* | c.1867G>T | p.V623L | 37.8% |  |
|  |  |  |  |  |  |  | *SETBP1* | c.2602G>A | p.D868N | 37.5% |  |
| aCML#6 | M | 78 | 6.7 g/dl | 33.0 x 10^3^ | 9.0% |  | *ETNK1* | c.731A>G | p.N244S | 45.0% |  |
|  |  |  |  |  |  |  | *EZH2* | c.1820A>G | p.Y607C | 95.4% |  |
|  |  |  |  |  |  |  | *IDH2* | c.419G>A | p.R140Q | 44.3% |  |
|  |  |  |  |  |  |  | *NRAS* | c.35G>A | p.G12D | 40.5% |  |
| aCML#8 | M | 86 | n.d. | 30.0 x 10^3^ | 2.0% |  | *SETBP1* | c.2602G>T | p.D868Y | 45.4% |  |
|  |  |  |  |  |  |  | *SRSF2* | c.278_301del | p.P95_R102del | 42.4% |  |
| aCML#9 | M | 79 | n.d. | 70.0 x 10^3^ | 10.0% | <5% | *BRAF* | c.1790T>A | p.L597Q | 5.9% |  |
|  |  |  |  |  |  |  | *KRAS* | c.108A>G | p.I36M | 34.3% |  |
| aCML#10 | M | 74 | 9.8 g/dl | 46.0 x 10^3^ | 1.0% | ~20% | *NRAS* | c.35G>A | p.G12D | 5.9% | in transformation |
|  |  |  |  |  |  |  | *JAK2* | c.1849G>T | p.V617F | 4.0% |  |
| aCML#11 | M | 67 | 11.5 g/dl | 50.0 x 10^3^ | 2% |  | *ASXL1* | c.2539insT | p.S847Ffs*47 | 44.9% |  |
|  |  |  |  |  |  |  | *CSF3R* | c.1853C>T | p.T618I | 37.5% |  |
|  |  |  |  |  |  |  | *CSF3R* | c.2296C>T | p.Q766* | 29.9% |  |
|  |  |  |  |  |  |  | *SETBP1* | c.2602G>A | p.D868N | 48.6% |  |
|  |  |  |  |  |  |  | *SRSF2* | c.284C>A | p.P95H | 52.1% |  |
| aCML#12 | W | 75 | 6.1 g/dl | 95.0 x 10^3^ | 6.0% | >5% | *CSF3R* | c.1853C>T | p.T618I | 31.1% |  |
|  |  |  |  |  |  |  | *SRSF2* | c.284C>G | p.P95R | 45.7% |  |
|  |  |  |  |  |  |  | *TET2* | c.3404G>A | p.C1135Y | 83.2% |  |
|  |  |  |  |  |  |  | *TP53* | c.832C>T | p.P278S | 32.3% |  |
| aCML#13 | W | 78 | 9.6 g/dl | 17.4 x 10^3^ | 7.0% | ~10% | *BRAF* | c.1790T>A | p.L597Q | 6.7% |  |
|  |  |  |  |  |  |  | *CBL* | c.1139T>C | p.L380P | 32.9% |  |
|  |  |  |  |  |  |  | *SETBP1* | c.2620G>A | p.D874N | 45.8% |  |
|  |  |  |  |  |  |  | *SRSF2* | c.284C>A | p.P95H | 51.0% |  |
| aCML#14 | M | 46 | n.d. | n.d. | 3.0% | >5% | *SETBP1* | c.2608G>A | p.G870S | 48.4% |  |
|  |  |  |  |  |  |  | *SRSF2* | c.278_301del | p.P95-R102del | 43.5% |  |
| aCML#15 | W | 53 | 13.7 g/dl | 25.5 x 10^3^ | 2.0% | <5% | *NRAS* | c.35G>A | p.G12D | 35.7% |  |
|  |  |  |  |  |  |  | *NRAS* | c.37G>A | p.G13C | 9.0% |  |
|  |  |  |  |  |  |  | *SETBP1* | c.2602G>A | p.D868N | 48.8% |  |
|  |  |  |  |  |  |  | *SRSF2* | c.284C>A | p.P95H | 57.1% |  |
| aCML#16 | M | 60 | n.d. | n.d. | n.d. |  | *NRAS* | c.34G>A | p.G12S | 24.2% |  |
|  |  |  |  |  |  |  | *NRAS* | c.182A>G | p.Q61R | 19.1% |  |
|  |  |  |  |  |  |  | *SETBP1* | c.2602G>A | p.D868N | 47.7% |  |
|  |  |  |  |  |  |  | *SRSF2* | c.284C>T | p.P95L | 36.7% |  |
| aCML#17 | M | 79 | 8.6 g/dl | 69.3 x 10^3^ | 3.0% | <5% | *CSF3R* | c.1853C>T | p.T618I | 42.0% |  |
|  |  |  |  |  |  |  | *SRSF2* | c.284C>G | p.P95R | 42.3% |  |
| aCML#18 | W | 81 | 11.1 g/dl | 89.2 x 10^3^ | 6.0% | >5% | *ASXL1* | c.2182G>T | p.Q728* | 39.6% |  |
|  |  |  |  |  |  |  | *DNMT3A* | c.2644C>T | p.R882C | 46.2% |  |
| aCML#19 | M | 61 | 6.6 g/dl | 24.4 x 10^3^ | 9.7% |  | *TET2* | c.1850del | p.R617Sfs*19 | 46.4% |  |
|  |  |  |  |  |  |  | *RUNX1* | c.822_825delACCA | p.Q274Hfs*36 | 33.5% |  |
| aCML#20 | M | 75 | 13.4 g/dl | n.d. | 1.2% |  | *KRAS* | c.202C>T | p.R68W | 45.7% |  |
|  |  |  |  |  |  |  | *SETBP1* | c.2608G>A | p.G870S | 73.0% |  |
|  |  |  |  |  |  |  | *SRSF2* | c.284C>A | p.P95H | 54.1% |  |
|  |  |  |  |  |  |  | *TET2* | c.3379C>T | p.Q1127* | 51.1% |  |
| aCML#22 | M | 75 | n.d. | n.d. | n.d. |  | *ASXL1* | c.1888_1910del23 | p.E635Rfs*15 | 21.4% |  |
|  |  |  |  |  |  |  | *CBL* | c.1223G>C | p.W408S | 11.2% |  |
|  |  |  |  |  |  |  | *FLT3* | c.2503G>T | p.D835Y | 4.2% |  |
|  |  |  |  |  |  |  | *KRAS* | c.74A>G | p.Q25R | 43.5% |  |
|  |  |  |  |  |  |  | *NRAS* | c.35G>A | p.G12D | 5.0% |  |
|  |  |  |  |  |  |  | *SETBP1* | c.2608G>A | p.G870S | 39.1% |  |
|  |  |  |  |  |  |  | *SRSF2* | c.284C>T | p.P95L | 37.6% |  |
| aCML#23 | M | 50 | 14.3 g/dl | 23.0 x 10^3^ | n.d. |  | *ASXL1* | c.2197C>T | p.Q733* | 47.6% |  |
|  |  |  |  |  |  |  | *SETBP1* | c.2602G>A | p.D868N | 47.8% |  |
|  |  |  |  |  |  |  | *SRSF2* | c.284C>A | p.P95H | 54.2% |  |
| aCML#24 | M | 77 | 11.9 g/dl | 37.0 x 10^3^ | 2.0% |  | *EZH2* | c.863G>A | p.R288Q | 46.2% |  |
|  |  |  |  |  |  |  | *EZH2* | c.2069G>A | p.R690H | 48.5% |  |
|  |  |  |  |  |  |  | *SETBP1* | c.2608G>A | p.G870S | 42.3% |  |
|  |  |  |  |  |  |  | *TET2* | c.4075C>T | p.R1359C | 43.6% |  |
| aCML#25 | W | 80 | 8.4 g/dl | 29.6 x 10^3^ | 1.0% |  | *EZH2* | c.1978G>A | p.G660R | 50.2% |  |
|  |  |  |  |  |  |  | *IDH2* | c.419G>A | p.R140Q | 47.5% |  |
| aCML#27 | M | 86 | 13.7 g/dl | 15.7 x 10^3^ | 2.0% | >5% | *CSF3R* | c.1853C>T | p.T618I | 5.3% |  |
|  |  |  |  |  |  |  | *ETNK1* | c.731A>G | p.N244S | 48.0% |  |
|  |  |  |  |  |  |  | *EZH2* | c.1876G>A | p.V626M | 46.6% |  |
|  |  |  |  |  |  |  | *EZH2* | c.434T>G | p.F145C | 45.3% |  |
|  |  |  |  |  |  |  | *TP53* | c.658T>C | p.Y220H | 47.0% |  |
| aCML#28 | M | 77 | 8.8 g/dl | 20.0 x 10^3^ | 7.0% | >5% | *ASXL1* | c.2113G>T | p.E705* | 46.4% |  |
|  |  |  |  |  |  |  | *KRAS* | c.35G>T | p.G12V | 4.3% |  |
|  |  |  |  |  |  |  | *SRSF2* | c.284C>A | p.P95H | 52.3% |  |
|  |  |  |  |  |  |  | *RUNX1* | c.314_315delTG | p.V105Dfs*5 | 44.0% |  |
| aCML#29 | W | 64 | 13.5 g/dl | 38.2 x 10^3^ | 6.0% | >5% | *ASXL1* | c.2212G>T | p.G738* | 46.9% |  |
|  |  |  |  |  |  |  | *SRSF2* | c.284C>A | p.P95H | 50.3% |  |
