# Supplementary material for: Comprehensive mutation profiling and mRNA expression analysis in atypical chronic myeloid leukemia in comparison with chronic myelomonocytic leukemia
Source: Cancer Med. 2019 Jan 11;8(2):742–50. doi: 10.1002/cam4.1946 (PMC6382710; doi:10.1002/cam4.1946)
Supplement: Supplementary file 6 [file CAM4-8-742-s006.docx]

Supplementary Table S4 Available clinical data and mutation profiling results of the CMML patient sample cohort (n=59). Available biopsies of CMML patients sometimes show lower absolute monocyte counts than 1000 due to therapeutic effects. At the time of diagnosis, absolute monocyte count was above 1 x 10^9^/L in every case regarding to the WHO criteria.

| **Patient No.** | **Sex** | **Age** | Hemoglobin | **Leucocytes** | **Monocytes** | **Blasts** | **Gene mutations** | **Coding** | **Protein** | **Allelic frequency** | **Comment** |
| --- | --- | --- | --- | --- | --- | --- | --- | --- | --- | --- | --- |
| CMML#1 | W | 77 | 12.0 g/dl | 32.7 x 10^3^ | 4.9% | <20% | *SRSF2* | c.284C>G | p.P95R | 48.7% |  |
| CMML#2 | M | 82 | 11.4 g/dl | 8.3 x 10^3^ | 18.0% |  | *ASXL1* | c.2077C>T | p.R693* | 44.4% |  |
|  |  |  |  |  |  |  | *SRSF2* | c.284C>A | p.P95H | 54.0% |  |
|  |  |  |  |  |  |  | *TET2* | c.822delC | p.N275Ifs*18 | 47.2% |  |
|  |  |  |  |  |  |  | *TET2* | c.3812_3813insG | p.C1271Wfs*29 | 45.3% |  |
| CMML#3 | W | 88 | 9.5 g/dl | 6.6 x 10^3^ | 20.0% |  | *NRAS* | c.35G>T | p.G12V | 19.5% |  |
|  |  |  |  |  |  |  | *TET2* | c.3587delT | p.R1197Gfs*50 | 43.7% |  |
|  |  |  |  |  |  |  | *TET2* | c.3765C>A | p.Y1255* | 42.9% |  |
| CMML#4 | M | 77 | 9.6 g/dl | 7.3 x 10^3^ | 5.7% |  | *SRSF2* | c.284C>G | p.P95R | 44.3% |  |
| CMML#5 | M | 71 | n.d. | 9.7 x 10^3^ | 15.0% |  | *TET2* | c.2374_2375insC | p.S794fs*8 | 49.6% |  |
|  |  |  |  |  |  |  | *TET2* | c.4079T>A | p.1360Q | 41.9% |  |
| CMML#6 | M | 81 | 7.4 g/dl | 8.9 x 10^3^ | 6.0% |  | *TET2* | c.2025_2026insA | p.L676Tfs*5 | 45.% |  |
|  |  |  |  |  |  |  | *TET2* | c.2596C>T | p.Q866* | 45.8% |  |
| CMML#7 | M | 70 | 9.1 g/dl | 15.8 x 10^3^ | 8.5% | >5% | *CBL* | c.1096G>A | p.E366K | 75.3% |  |
|  |  |  |  |  |  |  | *U2AF1* | c.101C>T | p.S34F | 43.9% |  |
| CMML#8 | M | 77 | 8.2 g/dl | 3.2 x 10^3^ | 40.9% |  | *TET2* | c.783delC | p.C262Vfs*31 | 80.6% |  |
| CMML#9 | M | 80 | 9.7 g/dl | 5.4 x 10^3^ | 30.3% | ~10% | *SRSF2* | c.284C>T | p.P95L | 41.9% |  |
|  |  |  |  |  |  |  | *TET2* | c.2785delC | p.P929Lfs*24 | 45.1% |  |
|  |  |  |  |  |  |  | *TET2* | c.3025C>T | p.Q1009* | 43.7% |  |
| CMML#10 | M | 90 | 10.5 g/dl | 26.1 x 10^3^ | 11.0% |  | *DNMT3A* | c.2644C>T | p.R882H | 45.5% |  |
|  |  |  |  |  |  |  | *NRAS* | c.35G>A | p.G12D | 4.4% |  |
|  |  |  |  |  |  |  | *NRAS* | c.175G>A | p.A59T | 10.2% |  |
|  |  |  |  |  |  |  | *TET2* | c.5482C>T | p.Q1828* | 46.7% |  |
| CMML#11 | M | 80 | 13.0 g/dl | 12.8 x 10^3^ | 18.0% |  | *SRSF2* | c.284C>A | p.P95H | 52.8% |  |
|  |  |  |  |  |  |  | *TET2* | c.3812_3813insG | p.C1271Wfs*29 | 46.9% |  |
|  |  |  |  |  |  |  | *TET2* | c.5074A>T | p.K1692* | 48.4% |  |
| CMML#12 | M | 74 | 9.0 g/dl | 5.7 x 10^3^ | 14.5% |  | *ASXL1* | c.3856C>T | p.Q1286* | 43.4% |  |
|  |  |  |  |  |  |  | *SRSF2* | c.284C>T | p.P95L | 39.7% |  |
|  |  |  |  |  |  |  | *TET2* | c.1204delT | p.S402Hfs*25 | 35.6% |  |
| CMML#13 | M | 80 | 10.9 g/dl | 13.8 x 10^3^ | 33.0% |  | *SETBP1* | c.2602G>A | p.D868N | 46.6% |  |
|  |  |  |  |  |  |  | *SRSF2* | c.284C>A | p.P95H | 50.1% |  |
|  |  |  |  |  |  |  | *SRSF2* | c.317C>A | p.P106Q | 51.0% |  |
|  |  |  |  |  |  |  | *TET2* | c.2855_2856insT | p.R953Kfs*19 | 47.4% |  |
| CMML#14 | M | 76 | 9.3 g/dl | 14.9 x 10^3^ | 38.5% |  | *SRSF2* | c.284C>A | p.P95H | 50.3% |  |
|  |  |  |  |  |  |  | *TET2* | c.2626_2627insG | p.Q876Afs*25 | 26.0% |  |
|  |  |  |  |  |  |  | *TET2* | c.2641_2642insA | p.R881fs*20 | 26.7% |  |
| CMML#15 | M | 73 | 12.5 g/dl | 7.1 x 10^3^ | 28.7% |  | *SRSF2* | c.284C>A | p.P95H | 44.8% |  |
|  |  |  |  |  |  |  | *TET2* | c.4133_4134insG | p.C1379Vfs*43 | 49.1% |  |
| CMML#16 | M | 78 | n.d. | n.d. | 42.0% |  | *CBL* | c.1186T>C | p.C396R | 4.4% |  |
|  |  |  |  |  |  |  | *TET2* | c.3183delA | p.V1062fs*4 | 39.6% |  |
|  |  |  |  |  |  |  | *TET2* | c.3732_3733delCT | p.Y1245Lfs*22 | 45.1% |  |
| CMML#17 | M | 87 | 7.9 g/dl | 8.6 x 10^3^ | n.d. |  | *SRSF2* | c.284C>A | p.P95H | 14.5% |  |
|  |  |  |  |  |  |  | *TET2* | c.1669C>T | p.Q557* | 45.0% |  |
|  |  |  |  |  |  |  | *TET2* | c.5570_5580del | p.P1857fs*14 | 24.8% |  |
| CMML#18 | M | 82 | 11.5 g/dl | 4.3 x 10^3^ | 29.2% |  | *TET2* | c.1993delA | p.T665Qfs*35 | 47.0% |  |
|  |  |  |  |  |  |  | *U2AF1* | c.470A>G | p.Q157R | 44.1% |  |
| CMML#19 | M | 81 | 10.1 g/dl | 4.7 x 10^3^ | 24.2% |  | *IDH1* | c.356G>A | p.R119Q | 48.0% |  |
|  |  |  |  |  |  |  | *TET2* | c.4757C>G | p.S1586* | 42.9% |  |
| CMML#20 | M | 81 | 11.4 g/dl | 13.0 x 10^3^ | 29.0% |  | *SRSF2* | c.284C>A | p.P95H | 48.1% |  |
|  |  |  |  |  |  |  | *TET2* | c.1837_1838insG | p.L615Afs*23 | 44.3% |  |
|  |  |  |  |  |  |  | *TET2* | c.4354C>T | p.R1452* | 48.6% |  |
| CMML#21 | M | 75 | 11.0 g/dl | 8.6 x 10^3^ | 21.0% |  | *ASXL1* | c.2141delC | p.R715fs*10 | 38.7% |  |
|  |  |  |  |  |  |  | *TP53* | c.824G>A | p.C275Y | 38.0% |  |
|  |  |  |  |  |  |  | *U2AF1* | c.476_477insGTATGA | p.E159_M160insYE | 33.7% |  |
| CMML#22 | M | 72 | n.d. | n.d. | n.d. | >5% | *KRAS* | c.35G>A | p.G12D | 14.8% |  |
|  |  |  |  |  |  |  | *SRSF2* | c.284C>A | p.P95H | 21.4% |  |
|  |  |  |  |  |  |  | *TET2* | c.3413delA | p.I1139fs*13 | 24.6% |  |
|  |  |  |  |  |  |  | *TET2* | c.4544_4545insT | p.A1516Sfs*9 | 64.4% |  |
| CMML#23 | W | 76 | 12.2 g/dl | 36.4 x 10^3^ | 19.0% |  | *ASXL1* | c.4238T>A | p.L1413* | 47.5% |  |
|  |  |  |  |  |  |  | *JAK2* | c.1849G>T | p.V617F | 25.0% |  |
|  |  |  |  |  |  |  | *SRSF2* | c.284C>A | p.P95H | 46.9% |  |
|  |  |  |  |  |  |  | *TET2* | c.2896_2897insTG | p.Q966fs*42 | 96.0% |  |
| CMML#24 | M | 83 | 5.0 g/dl | 200.0 x 10^3^ | n.d. | ~5% | *EZH2* | c.2035G>A | p.V679M | 45.0% |  |
|  |  |  |  |  |  |  | *FLT3* | c.2504A>T | p.D835V | 10.1% |  |
|  |  |  |  |  |  |  | *NRAS* | c.38G>A | p.G13D | 12.6% |  |
|  |  |  |  |  |  |  | *NRAS* | c.182A>G | p.Q61P | 19.3% |  |
|  |  |  |  |  |  |  | *TET2* | c.4271C>G | p.S1424* | 50.5% |  |
| CMML#25 | W | 80 | 10.3 g/dl | 4.2 x 10^3^ | 21.0% |  | *TET2* | c.3139_3140insA | p.T1047Nfs*11 | 42.3% |  |
| CMML#26 | M | 81 | 7.9 g/dl | 28.5 x 10^3^ | 12.0% | <20% | *EZH2* | c.434T>G | p.F145C | 91.9% |  |
|  |  |  |  |  |  |  | *SF3B1* | c.2098A>G | p.K700E | 48.5% |  |
|  |  |  |  |  |  |  | *TET2* | c.4481C>G | p.S1494* | 44.0% |  |
| CMML#27 | M | 80 | 7.0 g/dl | 10.0 x 10^3^ | 2.0% | ~10% | *DNMT3A* | c.2074del | p.Q692Rfs*13 | 34.0% | MF1 |
|  |  |  |  |  |  |  | *NPM1* | c.859_860insTCTG | p.W288Cfs*12 | 44.2% |  |
| CMML#28 | M | 75 | 12.2 g/dl | 15.3 x 10^3^ | n.d. |  | *ASXL1* | c.1773C>A | p.Y591* | 49.0% |  |
|  |  |  |  |  |  |  | *EZH2* | c.1954T>C | p.S652P | 93.2% |  |
|  |  |  |  |  |  |  | *RUNX1* | c.743delA | p.N248Tfs*6 | 41.1% |  |
|  |  |  |  |  |  |  | *TET2* | c.2236C>T | p.Q746* | 93.3% |  |
| CMML#29 | W | 79 | n.d. | n.d. | n.d. | <5% | *KRAS* | c.38G>C | p.G13A | 12.5% | MF1 |
|  |  |  |  |  |  |  | *SRSF2* | c.284C>A | p.P95H | 42.8% |  |
|  |  |  |  |  |  |  | *TET2* | c.3844_3846del | p.G1282del | 36.0% |  |
|  |  |  |  |  |  |  | *TET2* | c.4393C>T | p.R1465* | 47.2% |  |
| CMML#30 | W | 59 | 9.2 g/dl | 5.0 x 10^3^ | 25.0% | <5% | *No detectable pathogenic variants* | | | | |
| CMML#31 | M | 73 | 12.8 g/dl | 29.2 x 10^3^ | 30.4 % |  | *SRSF2* | c.280_281insCGC | p.R94dup | 37.4% |  |
|  |  |  |  |  |  |  | *TET2* | c.3058C>T | p.Q1020* | 88.7% |  |
| CMML#32 | M | 65 | 15.0 g/dl | 45.6 x 10^3^ | 14.6% | <20% | *RUNX1* | c.318G>C | p.W106C | 46.0% |  |
|  |  |  |  |  |  |  | *SRSF2* | c.284C>A | p.P95H | 49.3% |  |
| CMML#33 | W | 67 | 10.9 g/dl | 18.6 x 10^3^ | 15.0% | >10% | *SF3B1* | c.2098A>G | p.K700E | 44.4% |  |
|  |  |  |  |  |  |  | *TET2* | c.2263G>T | p.E755* | 44.2% |  |
|  |  |  |  |  |  |  | *TET2* | c.4074C>A | p.C1358* | 47.0% |  |
| CMML#34 | M | 69 | 11.3 g/dl | 0.7 x 10^3^ | 20.9% |  | *SETBP1* | c.2621A>T | p.D874V | 8.7% |  |
|  |  |  |  |  |  |  | *TET2* | c.1696G>T | p.E566* | 44.4% |  |
|  |  |  |  |  |  |  | *TET2* | c.4600C>T | p.Q1534* | 43.5% |  |
| CMML#35 | W | 81 | n.d. | 52 x 10^3^ | 23.0% | <5% | *NRAS* | c.34G>A | p.G12S | 29.0% |  |
|  |  |  |  |  |  |  | *U2AF1* | c.470A>G | p.Q157R | 43.6% |  |
| CMML#36 | M | 75 | 8.1 g/dl | 7.5 x 10^3^ | 24.6% | <20% | *CBL* | c.1112A>G | p.Y371C | 21.0% |  |
|  |  |  |  |  |  |  | *CBL* | c.1202G>T | p.C401F | 22.2% |  |
|  |  |  |  |  |  |  | *RUNX1* | c.341T>G | p.I114S | 44.5% |  |
|  |  |  |  |  |  |  | *SRSF2* | c.284C>T | p.P95L | 38.6% |  |
|  |  |  |  |  |  |  | *TET2* | c.4213G>T | p.E1405* | 42.5% |  |
| CMML#37 | M | 75 | 12.5 g/dl | 10.1 x 10^3^ | 15.0% |  | *NRAS* | c.35G>A | p.G12D | 35.4% |  |
|  |  |  |  |  |  |  | *NRAS* | c.176C>A | p.A59D | 6.1% |  |
|  |  |  |  |  |  |  | *SRSF2* | c.284C>G | p.P95R | 44.5% |  |
|  |  |  |  |  |  |  | *TET2* | c.2890C>T | p.Q964* | 47.9% |  |
|  |  |  |  |  |  |  | *TET2* | c.4954C>T | p.Q1652* | 52.2% |  |
| CMML#38 | M | 76 | 8.4 g/dl | 24.5 x 10^3^ | 14.0% |  | *NRAS* | c.35G>T | p.G12V | 24.9% |  |
|  |  |  |  |  |  |  | *SRSF2* | c.284C>A | p.P95H | 11.3% |  |
|  |  |  |  |  |  |  | *TET2* | c.3885C>G | p.Y1295* | 6.7% |  |
|  |  |  |  |  |  |  | *TET2* | c.4122C>G | p.C1374W | 81.8% |  |
| CMML#39 | M | 69 | n.d. | 30.0 x 10^3^ | 42.0% |  | *NRAS* | c.179G>A | p.G60E | 40.1% |  |
|  |  |  |  |  |  |  | *SRSF2* | c.284C>T | p.P95L | 34.0% |  |
|  |  |  |  |  |  |  | *TET2* | c.1449T>A | p.N483* | 92.0% |  |
|  |  |  |  |  |  |  | *TP53* | c.848G>A | p.R283H | 48.7% |  |
| CMML#40 | W | 71 | 12.2 g/dl | 4.1 x 10^3^ | 28.0% |  | *IDH2* | c.419G>A | p.R140Q | 26.8% |  |
|  |  |  |  |  |  |  | *SRSF2* | c.284C>T | p.P95L | 30.0% |  |
| CMML#41 | W | 75 | 8.4 g/dl | 186.2 x 10^3^ | 7.5% | <5% | *ASXL1* | c.1773C>A | p.Y591* | 48.9% |  |
|  |  |  |  |  |  |  | *EZH2* | c.2050C>T | p.R684C | 47.1% |  |
|  |  |  |  |  |  |  | *RUNX1* | c.780T>G | p.Y260* | 44.8% |  |
|  |  |  |  |  |  |  | *SRSF2* | c.284C>A | p.P95H | 49.3% |  |
|  |  |  |  |  |  |  | *TET2* | c.3785G>A | p.R1262Q | 48.2% |  |
|  |  |  |  |  |  |  | *TET2* | c.4076G>A | p.R1359H | 47.9% |  |
| CMML#42 | W | 81 | 14.4 g/dl | 4.3 x 10^3^ | 22.1% |  | *CALR* | c.1141_1143del | p.E381del | 52.6% |  |
|  |  |  |  |  |  |  | *SRSF2* | c.284C>G | p.P95R | 38.2% |  |
|  |  |  |  |  |  |  | *TET2* | c.2252_2253insA | p.N752Kfs*2 | 47.4% |  |
|  |  |  |  |  |  |  | *TET2* | c.3765C>A | p.Y1255* | 45.1% |  |
| CMML#43 | M | 77 | 14.0 g/dl | n.d. | 32.0% | >5% | *EZH2* | c.2143delA | p.I715Ffs*25 | 18.9% |  |
|  |  |  |  |  |  |  | *TET2* | c.4870C>T | p.Q1624* | 40.3% |  |
|  |  |  |  |  |  |  | *TET2* | c.5581G>A | p.G1861R | 44.7% |  |
| CMML#44 | W | 77 | n.d. | n.d. | n.d. | ~10% | *ASXL1* | c.1992_1993insC | p.S665fs*3 | 45.4% |  |
|  |  |  |  |  |  |  | *SETBP1* | c.2602G>A | p.D868N | 44.9% |  |
| CMML#45 | W | 81 | 14.0 g/dl | 14.4 x 10^3^ | 18.0% |  | *NRAS* | c.35G>A | p.G12D | 46.3% |  |
|  |  |  |  |  |  |  | *TET2* | c.2953delC | p.P985fs*22 | 49.5% |  |
|  |  |  |  |  |  |  | *TET2* | c.4690G>T | p.G1564* | 45.4% |  |
| CMML#46 | W | 81 | n.d. | n.d. | 32.0% | >5% | *TET2* | c.3571C>T | p.Q1191* | 41.8% |  |
|  |  |  |  |  |  |  | *TET2* | c.3778delA | p.N1260Ifs*6 | 43.6% |  |
| CMML#47 | M | 26 | 11.3 g/dl | 4.0 x 10^3^ | 40.0% |  | *TET2* | c.1648C>T | p.R550* | 46.2% |  |
|  |  |  |  |  |  |  | *TET2* | c.4081G>C | p.G1361R | 46.5% |  |
| CMML#48 | M | 64 | 8.3 g/dl | 33.5 x10^3^ | 18.0% | <20% | *No detectable pathogenic variants* | | | | |
| CMML#49 | M | 68 | 14.8 g/dl | 3.1 x 10^3^ | 32.0% |  | *SRSF2* | c.284C>T | p.P95L | 36.3% |  |
|  |  |  |  |  |  |  | *TET2* | c.2207C>G | p.S736* | 46.5% |  |
|  |  |  |  |  |  |  | *TET2* | c.5699T>A | p.V1900D | 43.4% |  |
| CMML#50 | M | 67 | 13.8 g/dl | 6.0 x 10^3^ | 10.0% |  | *RUNX1* | c.462G>C | p.Q154H | 44.3% |  |
|  |  |  |  |  |  |  | *SRSF2* | c.284C>T | p.P95L | 41.8% |  |
|  |  |  |  |  |  |  | *TET2* | c.1303delC | p.H435Tfs*12 | 58.5% |  |
| CMML#51 | W | 79 | n.d. | n.d. | n.d. |  | *NRAS* | c.35G>A | p.G12D | 44.5% |  |
|  |  |  |  |  |  |  | *TET2* | c.1771delC | p.Q591Sfs*10 | 48.0% |  |
| CMML#52 | W | 75 | 9.1 g/dl | 14.5 x 10^3^ | 8.0% | <5% | *KIT* | c.2447A>T | p.D816V | 37.0% |  |
|  |  |  |  |  |  |  | *SRSF2* | c.284C>A | p.P95H | 47.2% |  |
|  |  |  |  |  |  |  | *TET2* | c.4442delC | p.L1482Wfs*89 | 93.0% |  |
| CMML#53 | M | 80 | 11.6 g/dl | 7.5 x 10^3^ | 23.2% |  | *U2AF1* | c.101C>T | p.S34F | 43.2% | MF1 |
| CMML#54 | W | 80 | 7.7 g/dl | 14.2 x 10^3^ | 33.5% | ~10% | *ASXL1* | c.3005delC | p.S1003fs*21 | 45.5% |  |
|  |  |  |  |  |  |  | *SRSF2* | c.284C>A | p.P95H | 48.3% |  |
|  |  |  |  |  |  |  | *TET2* | c.1918C>T | p.Q640* | 46.1% |  |
| CMML#55 | M | 56 | 10.4 g/dl | 2.7 x 10^3^ | n.d. |  | *SRSF2* | c.284C>A | p.P95H | 23.8% |  |
|  |  |  |  |  |  |  | *TET2* | c.4272delT | p.D1425Tfs*23 | 12.7% |  |
| CMML#56 | M | 76 | 9.5 g/dl | 10.2 x 10^3^ | 31.0% | <5% | *RUNX1* | c.222delC | p.D75Tfs*47 | 50.4% |  |
|  |  |  |  |  |  |  | *TET2* | c.3305_3312del | p.N1103fs*24 | 41.2% |  |
| CMML#57 | W | 81 | 8.5 g/dl | 15.6 x 10^3^ | 6.6% |  | *SRSF2* | c.284C>T | p.P95L | 33.6% |  |
|  |  |  |  |  |  |  | *TET2* | c.3986T>G | p.L1329R | 40.5% |  |
|  |  |  |  |  |  |  | *TET2* | c.4767T>G | p.Y1589* | 44.7% |  |
| CMML#58 | M | 53 | 14.2 g/dl | 29.0 x 10^3^ | 25.0% | >5% | *DNMT3A* | c.2645G>A | p.R882H | 46.4% |  |
|  |  |  |  |  |  |  | *IDH1* | c.394C>T | p.R132C | 11.3% |  |
|  |  |  |  |  |  |  | *IDH2* | c.419G>A | p.R140Q | 36.5% |  |
|  |  |  |  |  |  |  | *SRSF2* | c.284C>T | p.P95L | 42.5% |  |
|  |  |  |  |  |  |  | *RUNX1* | c.341C>G | p.S114* | 42.5% |  |
| CMML#59 | M | 76 | 8.0 g/dl | 234.5 x 10^3^ | 12.5% | >5% | *EZH2* | c.619C>T | p.R207* | 44.0% |  |
|  |  |  |  |  |  |  | *TET2* | c.2757C>A | p.Y919* | 30.3% |  |
